# Treatment of Extra — Abdominal Desmoid Tumors with Chemotherapy

**DOI:** 10.3390/cancers3033394

**Published:** 2011-08-25

**Authors:** Corey Montgomery, Cynthia Emory, Sheila Adams, Jonathan Cohen, John David Pitcher, Benjamin Kyle Potter, H. Thomas Temple

**Affiliations:** 1 Rockefeller Cancer Institute, University of Arkansas for Medical Sciences, Little Rock, AR 72211, USA; E-Mail: comontgomery@uams.edu; 2 Wake Forest School of Medicine, Medical Center Blvds, Winston-Salem, NC 27157, USA; E-Mail: clemory@wakehealth.edu; 3 Department of Orthopaedics and Rehabilitation, University of Miami Miller School of Medicine, Cedars Medical Center, 1400 NW 12th Avenue (R-12), Miami, FL 33136, USA; E-Mail: sadams2@med.miami.edu; 4 Division of Psychology, University of Miami Miller School of Medicine, 1695 N.W. 9th Ave. (D-29), Miami, FL 33136, USA; E-Mail: jcohen@um-jmh.org; 5 Department of Orthopaedics and Rehabilitation, University of Miami School of Medicine, 1400 NW 12th Avenue (R-12), Miami, FL 33136, USA; E-Mail: Dpitcher@med.miami.edu; 6 Department of Orthopaedic Surgery, Walter Reed Army Medical Center, 6900 Georgia Avenue North West, Washington, D.C., 20307, USA; E-Mail: bkpotter@hotmail.com; 7 Department of Orthopaedic Surgery, University of Miami Miller School of Medicine, 1600 N.W. 10th Avenue (R-12), Miami, FL 33136, USA

**Keywords:** fibromatosis, desmoid tumors, chemotherapy, methotrexate, vinblastine, vinorelbine

## Abstract

Fibromatosis, or extra-abdominal desmoid tumor, is a benign disease which often has an aggressive clinical course that can be difficult to treat. We performed a retrospective review of 16 patients (12 females and four males) with a mean age of 34.2 years treated with methotrexate and vinblastine for newly diagnosed or recurrent extra-abdominal desmoid tumor. The mean age of our patient cohort was 34.2 years (range 11–70), and the mean tumor size was 11.5 cm (range 2.5–21.2 cm). The mean duration of therapy was 12 months with an average follow-up of 43 months (range 1–149 months). Fourteen of 16 patients demonstrated a clinical response to treatment. Eight of 14 patients demonstrated a radiologic decrease in tumor size. Only one patient progressed on therapy. Six patients developed recurrent symptoms after discontinuation of treatment. Chemotherapy-related symptoms including neutropenia, nausea, and vomiting were common and observed in most patients, however these side effects were mild and transient. Five patients developed peripheral neuropathy that prompted a change from vinblastine to vinorelbine during treatment. One potentially life-threatening complication (pneumocystis pneumonia) occurred which was diagnosed early and successfully treated. The use of methotrexate and vinblastine/vinorelbine in the management of fibromatosis appears to be an effective treatment with minimal treatment-related side effects.

## 1. Introduction

**Figure 1.**
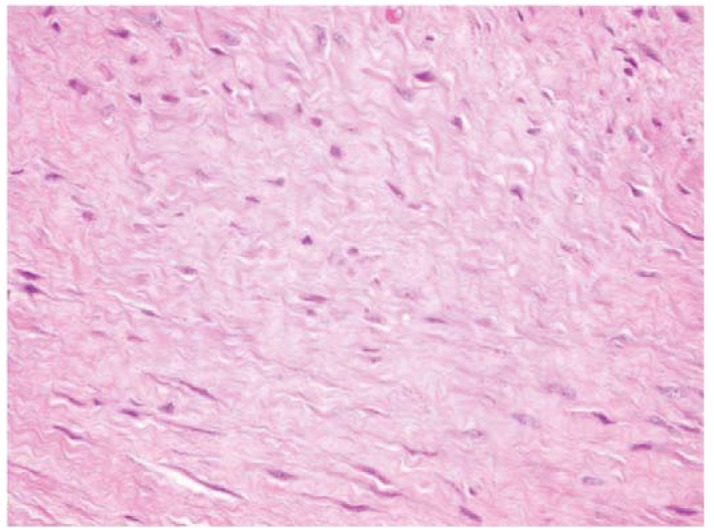
Histological appearance of fibromatosis.

Fibromatosis or extra-abdominal desmoid tumors are rare, benign tumors of fibroblastic origin (Figure 1) [1]. Though these tumors are benign, they can be locally aggressive resulting in significant morbidity and sometimes death when tumor invades vital organs [2-6]. The etiology of fibromatosis is unknown, but associations with trauma, sex hormones and chromosomal aberrations have been found [6-9]. These tumors have been classified based upon anatomic location as they can occur anywhere in the body yet maintain a consistent histologic appearance [1]. Superficial or fascial fibromatosis involve the palmar, plantar, penile and knuckle fascial layers. Deep or musculoaponeurotic fibromatosis is divided into extra-abdominal desmoid tumors (EADT) and intra-abdominal desmoid tumors (IADT). Although superficial and deep fibromatosis are histiologically similar in appearance they follow different clinical courses [1,6]. Given the locally aggressive nature of deep fibromatosis, surgical excision has been the principle method of treatment but it is sometimes combined with radiotherapy [10-15]. Morbidity of surgery and radiation in addition to disappointing oncologic results has generated interest in other potentially less morbid and more effective modalities. Recent reports have demonstrated success using medical therapy for patients with aggressive fibromatosis deemed unresectable or recurrent after previous unsuccessful traditional modalities (surgery and radiation) in adults and children [16-39]. Toxic and non-toxic agents are utilized in the medical management of deep fibromatosis. Non-steroidal anti-inflammatories (NSAIDS) and hormonal agents are examples of non-toxic agents employed in the medical management of fibromatosis. Several types of NSAIDS, including sulindac, indocin, and Cox-2 inhibitors, have been described in the literature [16-20]. Tsukada *et al.* reviewed their experience with sulindac in 14 patients [17] reporting one complete response and seven partial responses. Hormonal agents such as tamoxifen and toremifene have been used in the treatment of aggressive fibromatosis, but it is difficult to draw conclusions on these studies using hormonal agents since most are case reports with disparate results from complete responses to progress of disease [16,20,21]. Toxic agents such as doxorubicin, cyclophosphamide, vinblastine (VBL), vinorelbine (VNL) and methotrexate (MTX) have been prescribed in the treatment of aggressive fibromatosis [22-34]. Specifically, low dose MTX and VBL in combination have demonstrated greater than a 50% response rate in both pediatric and adult populations in most studies [22-28]. While chemotherapeutic agents such as doxorubicin and cyclophosphamide are reported to control local disease treatment-related toxicities mitigate widespread use [3,30-36]. Interferons and the tyrosine-kinase inhibitors such as, imantinib have been rarely reported in the treatment of aggressive fibromatosis with reported clinical successes [3,16,37-41].The purpose of the present study is to review the use of MTX and VBL in the treatment of EADT at a single institution. We hypothesize that the use of MTX and VBL will be effective in diminishing the clinical symptoms and the radiologic size of tumors with minimal treatment-related complications.

## 2. Materials and Methods

Sixteen patients with histologically confirmed EADT treated with MTX and VBL from 1990–2010 were retrospectively reviewed after institutional review board approval. All data were obtained from hospital medical records.

### Selection Criteria

Each patient was counseled on the various treatments options available for the management of fibromatosis. Patient's that failed previous treatments (non-operative and operative) and patients with tumors in locations where the surgeon determined considerable morbidity associated with excision were encouraged to consider chemotherapy.

Prior to initiation of therapy each patient had a documented complete history and physical examination, MRI of the anatomic location, complete blood count, platelet count, and basic metabolic panel. Once therapy was implemented, each patient was subsequently monitored weekly with routine blood tests and physical examinations to detect chemotherapy-related toxicities. Patients received 50 mg of MTX and 10 mg of VBL intravenously weekly, with dosing adjustments made as needed throughout treatment based on treatment-related toxicities. If patients were not able to return to our institution weekly for treatment, local medical oncologists were enlisted to administer the treatment protocol. Responses to therapy were measured clinically and radiologically. Patient's symptoms were categorized based upon a retrospective chart review of description of symptoms at the time of each visit. Symptoms prior to the start and at the conclusion of therapy determined the final clinical response. Clinical responses were documented as complete clinical response (all presenting symptoms resolved), partial clinical response (some of the presenting symptoms resolved), no response (symptoms stayed the same) or progression of disease (symptoms worsen). Any increase in the size of mass or worsening of symptoms related to the mass during the study period was defined as progression. Imaging responses were determined by using bidimensional measurements of the tumor on MRI every 3–6 months as either complete resolution (Grade 1), >50% decrease in size (Grade 2), <50% decrease in size (Grade 3), no progression of the tumor size (Grade 4) or progression of tumor size (Grade 5). Any increase in the size of the mass during the study period was defined as progression. Recurrence was defined as any symptom that abated during therapy and recurred after treatment or by radiographic tumor enlargement. Two orthopaedic oncologists independently reviewed the imaging studies to determine progression or recurrence. All chemotherapy related toxicities were recorded and treated accordingly. If neurotoxicities (numbness, tingling, *etc.*) due to VBL were observed during treatment, VBL was stopped and the patient was started on VNL 20 mg/m^2^ weekly [39]. This treatment protocol was continued until symptoms resolved, or until the patient elected to forego any further treatment because of toxicities, lack of clinical response, or personal choice.

## 3. Results

Sixteen patients were treated with the most common symptoms at presentation being pain and presence of a mass. Five patients had recurrent tumors at the initiation of treatment of which one had previous treatment with toremifene, one had previous treatment with tamoxifen and sulindac, two had previous surgical excision alone, and one had previous treatment with tamoxifen and surgical excision. No patients with recurrent tumor had received prior radiotherapy nor did any patient have familial adenomatous polyposis (Table 1). The mean follow-up was 43 months (range 1–149 months). Clinically, 14 patients responded to treatment at last follow-up, with four complete responses and ten partial responses. One patient had no response to therapy while one patient had progressive symptoms during treatment. Radiologically, 14 patients had pre- and post- imaging available for review at the time of analysis. Eight patients demonstrated a decrease in tumor size. One patient had a Grade 2 response and the remaining seven patients had a Grade 3 response. Of the remaining patients with imaging available for review, four patients had a Grade 4 response while only one patient had a Grade 5 while on therapy (Table 2). The mean duration of therapy was 12.1 months (range 1–39 months). Six patients developed recurrence of symptoms after discontinuation of therapy at an average of 25.6 months. The overall progression free Kaplan-Meier survival rate was 58% at five years of follow-up. Two patients resumed chemotherapy, two were treated with surgery and chemotherapy and two patients underwent surgery alone. The two patients treated with chemotherapy alone had resolution of their symptoms after restarting therapy. One of these patients recurred three times after stopping therapy (therapy stopped once due to pregnancy), but responded each time therapy was restarted. Two patients with recurrences had failed previous surgical management with one of these patients ultimately requiring an amputation for local disease control.

Chemotherapy related symptoms were common. The most common complication of chemotherapy was neutropenia followed by nausea/vomiting. One patient required cessation of chemotherapy after one month due to chronic diarrhea that developed after initiation of therapy resulting in this patient having only one month of follow-up. This patient elected to have no further treatments. 5 patients developed neurotoxicities related to VBL which required a change in treatment from VBL to VNL. One potentially life-threatening complication, pneumocysti pneumonia (PCP), developed during treatment, which was successfully treated with intravenous antibiotics and respiratory therapy. To minimize additional complications due to PCP 11 patients in the cohort subsequently received prophylactic sulfamethoxazole/trimethoprim and 12 patients were treated with leucovorin for prophylaxis of MTX-related stomatitis. No other cases of PCP were encountered, but other MTX related toxicities continued.

## 4. Discussion

**Figure 2.**
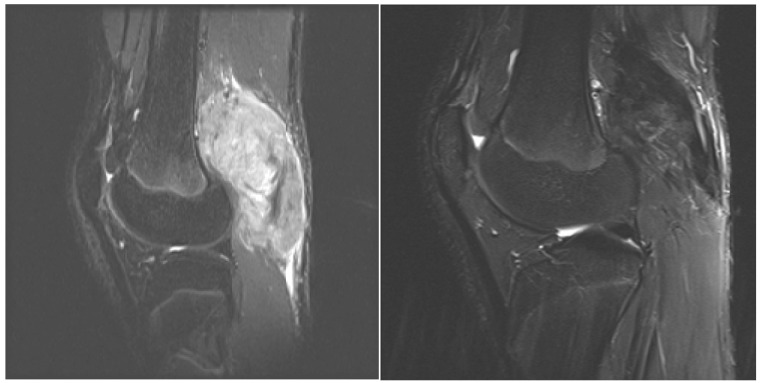
T2 sagittal MRI images before and after treatment demonstrate significant reduction in lesion size after treatment.

Over the past several years, there has been increased interest and utilization of medical management in the treatment of aggressive fibromatosis given the associated morbidity and high rate of local recurrence with surgery alone as well as combined surgery and radiotherapy. Medical management of EADT is highly variable as no widely accepted treatment has been established in the literature. This is a retrospective review of a medical protocol from a single institution utilized in the management EADT in sixteen patients. The medical protocol was originally described by Weiss and Lackman [22]. Modifications were made to the protocol by replacing VBL with VNL when neurotoxicity was detected in addition to using sulfamethoxazole/trimethoprim as prophylaxis for treatment related pneumocystis and leucovorin MTX-related toxicities. The findings in our study demonstrate efficacy using MTX and VBL in treatment of EADT in both pediatric and adult patients with 14 of 16 patients demonstrating a positive clinical response. These findings are consistent with previous reports [22,23,26,28]. Each of these studies demonstrated >50% response rate of either stabilization of disease or decrease in tumor size and/or symptoms using this regimen. Each study varied in their determination of response to treatment. Weiss and Lackman [22] reported on their experience with MTX and VBL in reporting two complete responses and four partial responses in adult patients using a combined assessment of clinical exam and imaging. Skapec *et al.* [25] reported in a prospective study of 26 children, 18 patients with either stable disease or a decrease in symptoms or tumor size using imaging to assess response. Azzarelli *et al.* [24] reported 40% of patients with a favorable radiologic response to therapy; however, up to 60% of the patients had tumor stabilization or minor tumor shrinkage. We choose to evaluate our treatment responses both radiologically as well as clinically. In our analysis, the radiographic response was not as robust as the clinical response. This regimen appears to be effective in resolving clinical symptoms that in many cases are disabling, but less effective in decreassing tumor size. When comparing pre- and post- treatment MRI an important observation is the decrease in signal intensity on T2 pulse weighted sequences in post treatment MRI which we believe demonstrates cell drop out and increased collagen matrix. In addition, there is decreased perilesional edema that may be responsible for the significant presenting pain and rapid relief following initiation of therapy (Figure 2). Recurrence of clinical symptoms after cessation of therapy occurred in six patients. In the two patients treated with chemotherapy alone, both had resolution of their symptoms after resuming chemotherapy. Azzarelli *et al.* [24] discussed symptom recurrence after discontinuation of therapy and also noted response to treatment after restarting therapy. Chemotherapy-related toxicity occurred in all patients except one in this series. Neutropenia was also most common toxicity reported in our cohort and in three other studies [22,24,26]. During the early years of the protocol, one patient developed PCP, so sulfamethoxazole/trimethoprim was given prophylactically to 11 subsequent patients with no additional pneumocystis complications or antibiotic side effects. No other studies utilizing MTX and VBL report PCP as a complication of therapy, but Skapek et al. reported five of 27 patients in their series developing life-threatening toxicities. For patients treated with leucovorin, neutropenia and nausea/vomiting persisted in the majority of patients, but stomatitis was only reported in two. Neurotoxity associated with VBL was encountered in 5 patients requiring a switch from VBL to VNL, but no change in success of the regimen was noted. Consideration is being given to treating all patients first-line with VNL since the patients switched from VBL to VNL in our study continued to respond well after crossover. The symptoms abated upon switching the patients from VBL to VNL.

When comparing combination chemotherapy to other combination chemotherapy regimens reported in literature, MTX and VBL/VNL was demonstrated in our study to have a very effective response rate without some of the known toxicities of some of the other regimens (Table 3).

Our study findings are limited given its retrospective nature and the limited number of patients, but we believe MTX and VBL/VNL are effective in the management of EADT in both children and adults. Toxicities associated with this regimen were overall mild and reversible. However, long periods of intravenous treatments may be prohibitive to implement as first-line treatment for all EADT. MTX and VBL should be considered for treatment of large, inoperable and aggressive recurrent EADT to control symptoms and stabilize disease progression with limited side effects.

## 5. Conclusions

The treatment of EADT with MTX and VBL/VNL rendered good clinical outcomes, but less satisfactory results radiologically. Our findings suggest that the use of MTX and VBL/VNL in the management of EADT may be an effective alternative to surgery, but longer follow-up with more patients will be necessary in order to establish the long-term outcomes of this treatment.

## Figures and Tables

**Table 1.** Demographic information for patients in the present study who received chemotherapy for treatment of fibromatosis.

**Patient**	**Age (years)**	**Sex**	**Location**	**Primary/Recurrence**	**Size (cm)**
1	22	F	Leg	P	19.3
2	11	F	Popliteal	P	9.7
3	24	F	Thigh	P	5.7
4	41	F	Torso	P	6.5
5	70	M	Shoulder	P	11
6	32	M	Thigh	P	6
7	50	F	Buttocks	P	5.7
8	62	F	Buttocks	R	14
9	34	F	Leg	P	9
10	40	F	Thigh	P	11
11	20	F	Torso	R	3.5
12	57	F	Shoulder	P	20
13	24	F	Thigh	R	16
14	33	M	Ankle	R	20
15	14	F	Thigh	P	21.2
16	13	M	Popliteal	P	8.6

C: Chemotherapy; S: Surgery; P: Primary; R: Recurrence.

**Table 2.** Duration and response to therapy, recurrence, treatment of recurrence and length of follow-up for patients receiving chemotherapy for treatment of fibromatosis in the present study.

**Patient**	**Total Duration of therapy (mo)**	**Response (Clinical)**	**Response (Radiologic)**	**Recurrence**	**Treatment of Recurrence**	**Follow-up**
1	28	Partial	Grade 3	Once	C	39
2	12	Stable	Grade 4	No		53
3	12	Partial	Grade 4	No		13
4	1	Partial		No		1
5		Partial	Grade 3	No		43
6	3	Partial	Grade 3	No		3
7	15	Complete	Grade 3	No		24
8	23	Partial	Grade 4	Once	C,S	149
9	7	Progress	Grade 5	Twice	S	61
10	11	Partial	Grade 2	No		24
11	47	Partial	Grade 3	Thrice	C	78
12	5	Partial		No		5
13	22	Complete	Grade 5	Once	C	69
14	28	Partial	Grade 4	Once	S	94
15	11	Complete	Grade 3	No		26
16	16	Partial	Grade 3	No		13

C: Chemotherapy, S: Surgery; Grade 1 (Complete), Grade 2 (>50% decrease in size), Grade 3 (<50% decrease in size), Grade 4 (stabilization), Grade 5 (Progression).

**Table 3.** Cytotoxic chemotherapy response rates of prior studies evaluating the effects of chemotherapy on fibromatosis.

**Author**	**Number of Patients**	**% Response**	**Type of Chemotherapy**
Raney *et al.* 1987 [35]	6	83	Vincristine, actinomycin, cyclophosphamide
Schnitzler *et al.* 1997 [32]	5	100	Doxrubicin, darcabazine, carboplatin
Klaase *et al.* 1989 [36]	7	43	Mephalan, doxorubicin
Patel *et al.* 1993 [33]	12	50	Doxrubicin, dacarbazine
Poritz *et al.* 2001 [31]	8	75	Doxrubicin, darcarbazine, carboplatin
Okuno and Edmonson 2003 [34]	7	85	Cyclophosphamide, doxorubicin, mitocyin cisplatin, ifosfamide, etoposide
Constantindiou *et al.* 2009 [30]	12	36	Pegylated liposomal doxrubicin

Percentage of response of chemotherapy was calculated base upon each individual study documentation of response (radiologic, clinical or both).
